# Taxonomy of the *Cryptopygus* complex. III. The revision of South African species of *Cryptopygus* and *Isotominella* (Collembola, Isotomidae)

**DOI:** 10.3897/zookeys.945.51860

**Published:** 2020-07-03

**Authors:** Mikhail B. Potapov, Charlene Janion-Scheepers, Louis Deharveng

**Affiliations:** 1 Senckenberg Museum of Natural History Görlitz, Am Museum 1, 02826, Görlitz, Germany Senckenberg Museum of Natural History Görlitz Görlitz Germany; 2 Moscow State Pedagogical University, Kibalchich str., 6, korp. 3, Moscow 129278, Russia Moscow State Pedagogical University Moscow Russia; 3 University of Cape Town, Department of Biological Sciences, Rondebosch, 7701, South Africa University of CapeTown Cape Town South Africa; 4 Iziko Museums of South Africa, 25 Queen Victoria Street, Cape Town, 8001, South Africa Iziko Museums of South Africa Cape Town South Africa; 5 Institut de Systématique, Évolution, Biodiversité ISYEB-UMR 7205-CNRS, MNHN, UPMC, EPHE, Muséum national d’Histoire naturelle, Sorbonne Universités, 45 rue Buffon, F-75005, Paris, France Sorbonne Universités Paris France

**Keywords:** endemism, South Africa, springtails, taxonomy

## Abstract

Species of the genera of the *Cryptopygus* complex in South Africa are morphologically revised. Five new species of the genus *Cryptopygus* Willem, 1902 **s. s.** and one new species of the genus *Isotominella* Delamare Deboutteville, 1948 are described. *Cryptopygus abulbus***sp. nov.** and *C. bulbus***sp. nov.** have only one chaeta on the anterior side of dens and no chaetae on the anterior side of manubrium, the latter species being characterized by the presence of a bulb at apex of antennae; *C. inflatus***sp. nov.** shows a rare combination of eight ocelli on each side of the head with a tridentate mucro; *C. longisensillus***sp. nov.** has five long s-chaetae on the fifth abdominal segment; *C. postantennalis***sp. nov.** is unique by having a very long and slender postantennal organ with strong inner denticles; *Isotominella laterochaeta***sp. nov.** is the second member of the genus and differs from the type species by many more anterior chaetae on the manubrium and the presence of chaetae on ventral side of metathorax. The genera are discussed and a key to all species of the *Cryptopygus* complex recorded in South Africa is given. The focus is on the Western Cape Province where the complex is the most diverse and sampling more complete than in other provinces of South Africa.

## Introduction

Although poor compared to Europe, the diversity of South Africa Collembola is increasingly better understood due to international collaborations since 2008 (Janion et al. 2011a, b, 2012; Janion-Scheepers et al. 2013, 2015; Potapov et al. 2011). The family Isotomidae, which include the dominant Collembola in soil and moss habitats of South Africa, has been particularly little studied, and currently consists of 19 confirmed described species (Janion-Scheepers et al. 2015). From samples collected over the last decade, especially in the Western Cape Province of South Africa, we found a large diversity of *Cryptopygus* species (Janion-Scheepers et al. 2011), which had not been recorded so far. Previous South African records of this genus include *Cryptopygus caecus* by Paclt (1959), now known as *Mucrosomia caeca* (Wahlgren, 1906), while Barra (1997) described *Cryptopygus riebi* Barra, 1997, now a synonym of *Pauropygus caussaneli* (Thibaud, 1996). We describe here the first five *Cryptopygus* species of South Africa and a new species of the genus *Isotominella*, monotypic up to this date. To summarize our data, we provide a key to all species of the *Cryptopygus* complex for South Africa. This is our second contribution to the taxonomy of the family Isotomidae of the country (Potapov et al. 2011) and the third piece of work on the *Cryptopygus* complex (Potapov et al. 2013; Potapov et al. 2017).

## Material and methods

Abbreviations:

**A, B, C, D, E** papillae of labial palp following Fjellberg (1999)

**Abd.** abdominal segments

**accp** s-chaeta(e) situated near or within p-row of chaetae

**Ant.** antennal segments

**bms** basal micro s-chaeta(e)

**Mac1, Mac2** Macrochaeta(e)

**MNHN** Muséum national d’Histoire Naturelle

**ms** micro s-chaeta(e) or ms-chaeta(e)

**MSPU** Moscow State Pedagogical University

**PAO** postantennal organ

**s** macro s-chaeta(e) or s-chaeta(e)

**SAMC** South African Museum, Cape Town

**SMNG** Senckenberg Museum of Natural History Görlitz

**Th.** thoracic segments.

Abbreviations on figures are given in associated legends. Nomenclature used follows Potapov (2001) and Fjellberg (1999).

Type material of the new species described below are deposited at the Iziko South African Museum, Cape Town (South Africa, **SAMC**), Senckenberg Museum of Natural History, Görlitz (Germany, **SMNG**), Moscow State Pedagogical University (Russia, **MSPU**), and Muséum national d’Histoire Naturelle, Paris (France, **MHNH**).

Specimens were mounted on cavity or flat microscope slides using Gisin’s solution or Marc André II mounting liquid, respectively. Specimens were studied using a Leica DM2500 microscope, while drawings were made using camera lucida.

## Taxonomic part

In our understanding, the *Cryptopygus* complex includes all taxa of Anurophorinae s. l. having Abd. V well separated from Abd. IV and fused with Abd. VI, and lacking any special apomorphy (e.g., presence of true spines at the end of abdomen, loss of PAO, furca, etc.). Both genera studied below belong to the *Cryptopygus* complex.

### Key to South African species of the *Cryptopygus* complex

**Table:** 

1	Abdominal tip with several foil-chaetae (chaetae with a cluster of cilia near the tip, Fig. 60)	***Hemisotoma thermophila* s. l. (Axelson, 1900)**
‒	Abdominal tip without foil-chaetae	**2**
2	S-chaetae in p-row of chaetae on Abd. I–III	**3**
‒	S-chaetae in mid-tergal position on Abd. I–III	**6**
3	Furca long: dens with more than 30 anterior chaetae	**4**
‒	Furca of medium size: dens with less than 20 anterior chaetae	**5**
4	Mucro falciform. Anterior side of manubrium with 4+4 chaetae. Without ocelli	***Arlea tridens* Barra, 1997**
‒	Mucro bidentate. Anterior side of manubrium with 1+1 chaetae. 1+1 ocelli	***Proisotomodes bipunctatus* (Axelson, 1903)**
5	Number of s-chaetae on Th. III-Abd. II 1/1,1. Pleural fold on mouth cone with two finger-like processes (fig. 20 in Potapov et al. 2013). Only on sea littoral	***Pauropygus caussaneli* (Thibaud, 1996)**
‒	Number of s-chaetae on Th. III-Abd. II 3/2,2. Pleural fold on mouth cone without finger-like processes (Fig. 52)	***Isotominella laterochaeta* sp. nov.**
6	Mucro with 2 teeth, dens not crenulated on posterior side	**7**
‒	Mucro with 3 or more teeth, dens crenulated on posterior side	**9**
7	Manubrium without chaetae on anterior side	**8**
‒	Manubrium with 1+1 chaetae on anterior side	***C. antarcticus* complex, several species**
8	Antennal segment IV with bulb (Figs 9, 10), tenaculum with one chaeta	***C. bulbus* sp. nov.**
‒	Antennal segment IV without bulb (Figs 17, 18), tenaculum with two chaetae	***C. abulbus* sp. nov.**
9	Blind, mucro with 3 teeth and 2 lateral basal spines	***Mucrosomia caeca* (Wahlgren, 1906)**
‒	With ocelli, mucro with 3 teeth and without lateral basal spines	**10**
10	PAO slender and long (twice as long as width of Ant. I), with strong inner denticles (Fig. 27)	***Cryptopygus postantennalis* sp. nov.**
‒	PAO normal (at most slightly longer than width of Ant. I), without strong inner denticles	**11**
11	Macrochaetae and s-chaetae on Abd. V short	***Cryptopygus inflatus* sp. nov.**
‒	Macrochaetae and s-chaetae on Abd. V long	***Cryptopygus longisensillus* sp. nov.**

#### Cryptopygus

Taxon classificationAnimaliaEntomobryomorphaIsotomidae

Willem, 1902

E1946AF1-B164-52BE-B5BA-66F462AF9B6C

##### Type species.

*Cryptopygus antarcticus* Willem, 1902.

##### Diagnosis.

A genus of the *Cryptopygus* complex. Medial s-chaetae in mid-tergal position from Th. II to Abd. III. Number of s-chaetae 4,3/2,2,2,3,5. Foil chaetae at the end of abdomen absent.

##### Discussion.

Here we follow our simplified characterisation of the genus proposed formerly (Potapov et al. 2017) that is an expanded version of diagnosis of Rusek (2002). The latter diagnosis offers to be within the limits of the definite number of ocelli (6+6), “*antarcticus*-like” furca and clavate tibiotarsal hairs. We presume a wide variation of the main characters: the mucro may be absent, bidentate or tridentate; the manubrium with or without anterior chaetae; the dens short and smooth to long and crenulated; the ocelli absent to their full set, i.e. 8+8; tenent tibiotarsal hairs clavate or pointed; number of ms-chaetae from 1,0/0,0,0 to 1,1/1,1,1; maxillary outer lobe with changeable shape of palp and number of sublobal hairs. In our view, the splitting of *Cryptopygus* to groups, subgenera or genera will be probably necessary, but calls for preliminary morphological revisions and expanded molecular data of known species.

Species closely related to *C. antarcticus*, i.e., belonging to *Cryptopygus* sensu Rusek, have also been found in this study in South Africa (see below) but their status remain unsolved because of the taxonomic and molecular complexity of this group (Deharveng, 1981; Stevens et al. 2006; McGaughran et al. 2010).

#### Cryptopygus bulbus sp. nov.

Taxon classificationAnimaliaEntomobryomorphaIsotomidae

6A1D4C35-0816-55CE-BE5B-441E88A98731

http://zoobank.org/BBDBE2CF-1565-4A84-A15F-5E51CB5C857E

Figures 1 , 2 , 4 , 6–14

##### Type material.

Holotype and thirteen paratypes: South Africa • Western Cape, Cederberg Wilderness Area, Wolfberg Crags; 32.471507S, 19.278397E; 19 Feb 2011; C. Janion-Scheepers leg.; litter, Tullgren extraction; RSA11_CED002, deposited at SAMC • six paratypes on four slides and seven paratypes in ethanol; same locality • sixteen paratypes; Western Cape, Cederberg Wilderness Area; 32.310167S, 19.175183E; Oct. 2008; C. Janion-Scheepers leg.; pitfall trap; RSA08_CED001. Four paratypes deposited in ethanol at SAMC, five paratypes on three slides deposited at SMNG, seven paratypes on three slides at MSPU.

##### Other material.

South Africa • Western Cape, Mont Rochelle Nature Reserve, Franschoek; 33.902967S, 19.158950E; 06 Oct. 2008; C. Janion-Scheepers leg.; *Erica* site with *Erica-Protea* (Ericaceae and Protacea) mixed litter, litter trap; MR644, MR648 • Western Cape, Kogelberg Nature Reserve; 34.328083S, 18.962250E; 29 Aug. 2008; C. Janion-Scheepers leg.; *Erica* Site, Litter trap (K467), with *Galenia* litter.

##### Diagnosis.

With a globular retractile bulb on Ant. IV. Organite on Ant. IV chili-like. 6+6 ocelli. Maxillary palp simple. Two sublobal hairs. Anterior side of manubrium without chaetae. Tenaculum with one chaeta.

##### Description.

**Figures 1–3.**
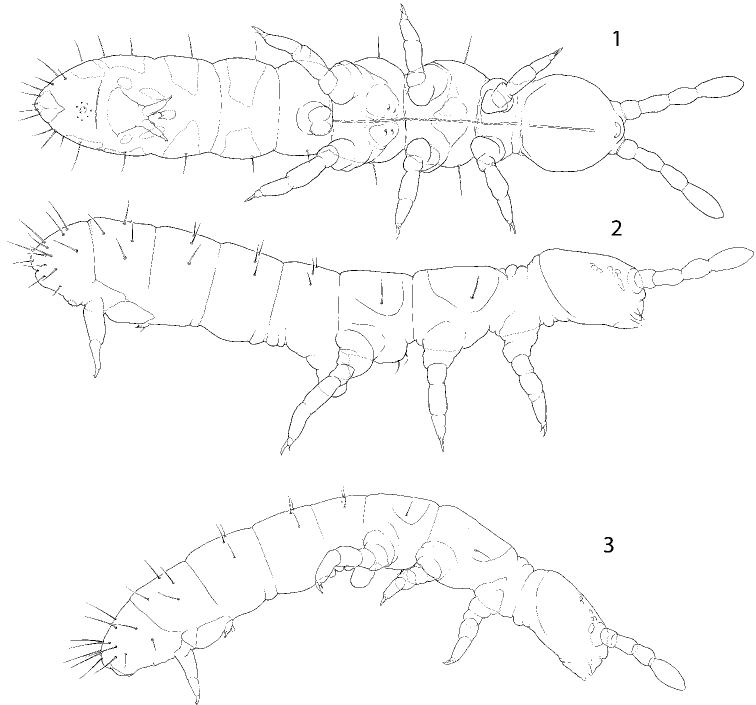
Appearance and macrochaetotaxy of *C. bulbus* sp. nov., ventral (**1**) and lateral (**2**) views, and *C. abulbus* sp. nov. (**3**).

**Figures 4–5.**
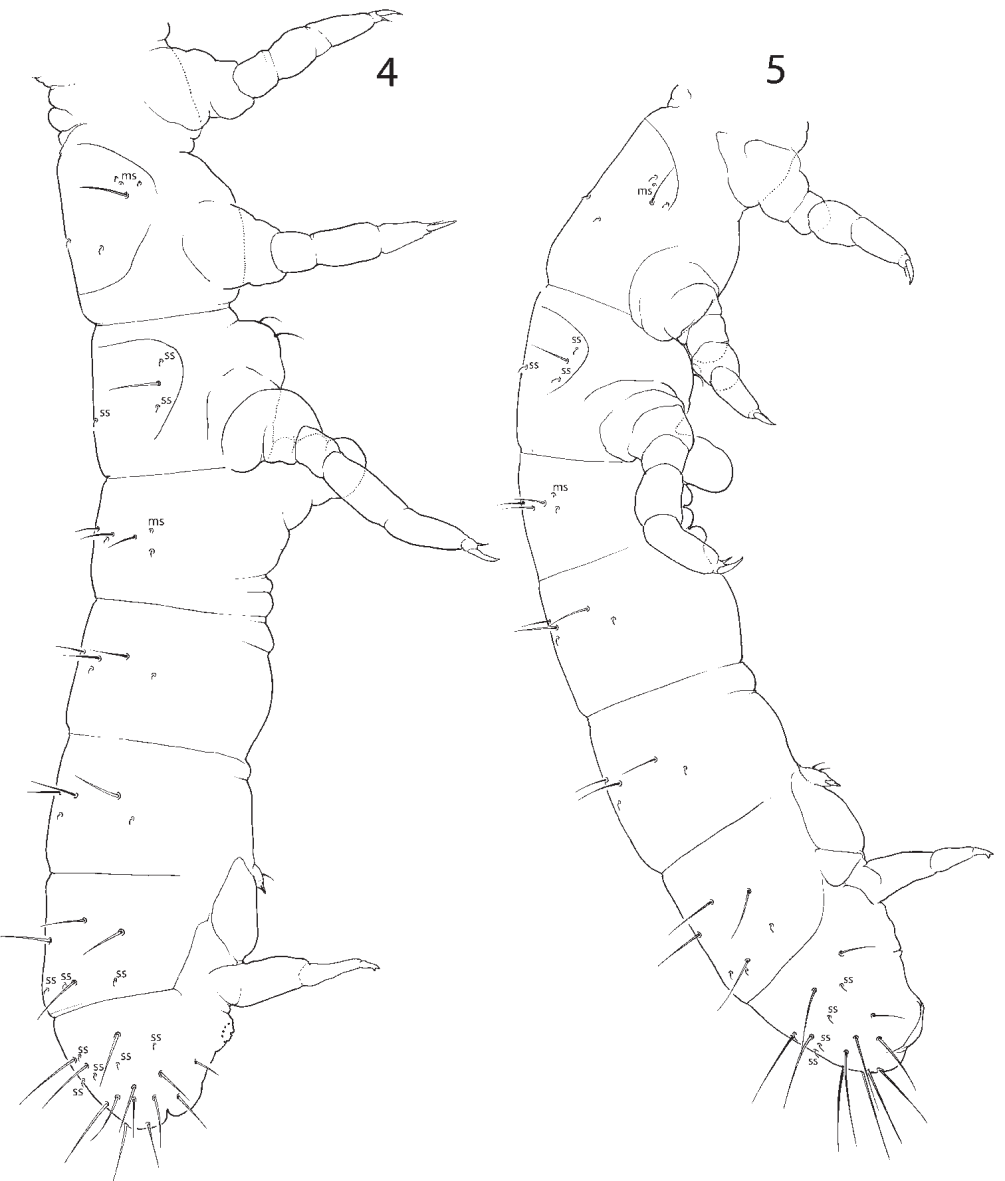
Macrochaetotaxy and s-ms-pattern of *C. bulbus* sp. nov. (**4**) and *C. abulbus* sp. nov. (**5**). Abbreviations: msms-chaeta, ss s-chaeta.

Body size 0.7–0.9 mm, habitus as in Figs 1, 2. Body with rather regular blue pigmentation, slender. Abd. V well separated from Abd. IV and fused with Abd. VI (Fig. 4). Cuticle “smooth”, with orthogonal primary granulation. Ocelli 6+6 arranged in anterior and posterior groups (Fig. 8), three in each. PAO more than twice as long as ocellus, 0.6–0.7 as long as width of Ant. I and 1.0–1.3 as long as inner unguis length. Maxillary head with unmodified lamellae. Maxillary outer lobe with two sublobal hairs, maxillary palp simple. Labral formula as 2/5,5,4, edge of labrum not reduced (Fig. 7). Labium with five usual papillae (*A–E*) and labial formula A1B3C0D4E6, guard chaetae e7 and b4 absent, three proximal and four basomedian chaetae. Ventral side of head with 4+4 chaetae. Ant. I with two ventral s-chaetae (s) and three small bms, two dorsal and one ventral, Ant. II with three bms and one latero-distal s, Ant. III with one bms and five distal s (including one lateral), without additional s-chaetae. S-chaetae on Ant. IV weakly differentiated. Organite long, of chili-like shape, set apart from subapical micro s-chaeta (Figs 9, 10). A globular retractile bulb embedded at tip of antennae, near pin-chaeta (Figs 9, 10).

**Figures 6–14.**
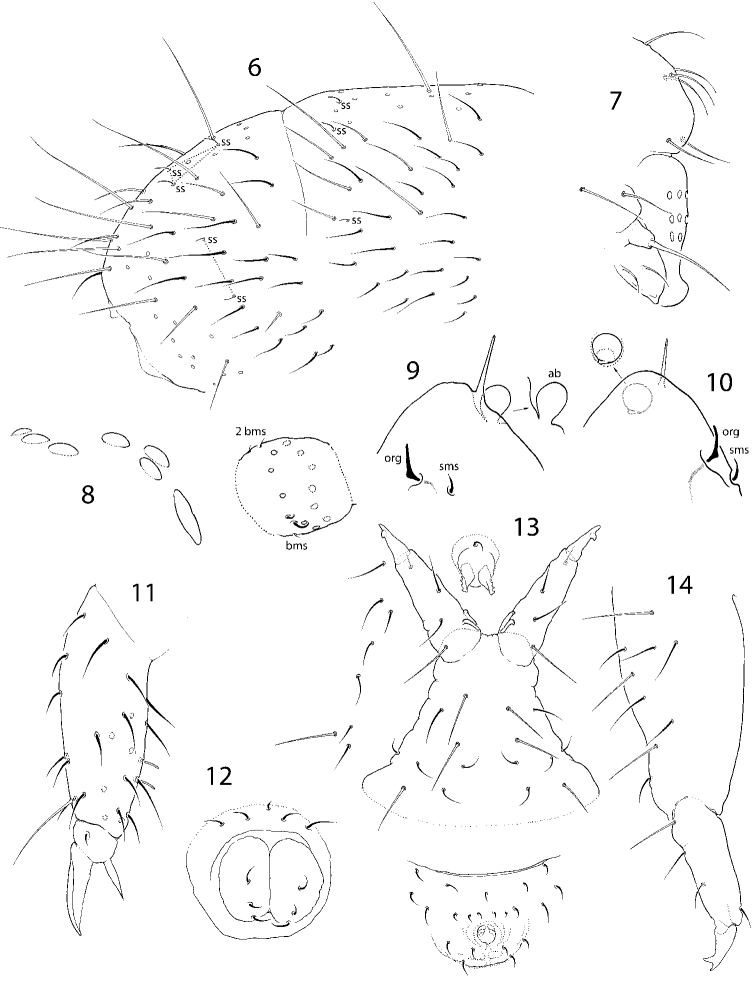
*C. bulbus* sp. nov. **6**Abd. IV and fused Abd. V and VI **7** labrum, lateral view **8** ocelli, PAO and Ant. I **9, 10** apical part of Ant. IV, different views **11** tibiotarsus and claw of Leg III in adult male **12** ventral tube, ventral view **13** furcal area, ventral view **14** furca, lateral view. Abbreviations: ab apical bulb, org organit, sms subapical ms-chaeta, bms basal ms-chaeta, ss s-chaeta.

Common chaetae often slightly serrated at the posterior part of Abd. V. S-formula as 4,3/2,2,2,3,5 (s), 1,0/1,0,0 (ms) (Fig. 7). Tergal s-chaetae much shorter than common chaetae and well distinguishable (Fig. 4). Medial s-chaetae on Th. II-Abd. III situated in mid-tergal position. On Abd. V, three dorsal s-chaetae (al, accp1, accp2) and two lateral ones slightly shorter (Fig. 6). Macrochaetae smooth and short, 1,1/3,3,3 in number, medial ones on Abd. VI 1.6–2.0 times longer than dens and 2.8–4.1 times longer than mucro. Foil chaetae at the tip of abdomen absent. Axial chaetotaxy as 5–6,4/3,3(4),3–4,4–6. Th. I and II without ventral chaetae, Th. III with 2+2 ventral chaetae (Fig. 1).

Unguis of normal shape, without teeth. Empodial appendage 0.5–0.7 as long as unguis. Tibiotarsi without additional chaetae on Leg I and II (21 chaetae), and with few ones on Leg III (>25), adult males with short thickened spurs on tibiotarsi III (Fig. 11). Tibiotarsal tenent hairs clavate, 1,2,2 on Tibiotarsi 1,2,3. Ventral tube with 3+3 laterodistal and 4–6 posterior chaetae, anteriorly without chaetae (Fig. 12). Tenaculum with 4+4 teeth and one chaeta. Anterior furcal subcoxa with 5–7, posterior one with three chaetae. Anterior side of manubrium without chaetae, posterior side with 4+4 laterobasal and 8–10 chaetae on main part, without lateral chaetae (Figs 13, 14). Dens short, without crenulation, with one rigid and short anterior and three posterior chaetae. Mucro bidentate. Ratio manubrium : dens : mucro = 2.5–4.1 : 1.7–2.2 : 1.

##### Etymology.

The name is derived from the presence of apical bulb on Ant. IV.

##### Distribution and ecology.

Currently known to occur in the southern part of the Western Cape Province of South Africa, including Kogelberg, Franschoek and the Table Mountain area (Cape Town). All specimens were collected from leaf litter in indigenous vegetation.

##### Discussion.

The species differs from other representatives of the *Cryptopygus* complex, if not from all Isotomidae of the Southern Hemisphere, by the presence of a globular bulb at tip of the antennae. The taxonomical value of this character is not fully clear. In the Northern Hemisphere several unrelated genera also possess an apical bulb which is embedded on the tip of the antennae, for example *Anurophorus* Nicolet, 1842 (most of the species), *Sibiracanthella* Potapov & Stebaeva, 1994, *Tuvia* Grinbergs, 1962 and *Vertagopus* Börner, 1906 (few species). The antennal bulb of *C. bulbus* sp. nov. is set apart from the apex, which is unlike in the aforementioned taxa. The only exception found was in specimens observed from Orangekloof (Cape Town), where the apical bulb was less developed.

For other differences of the new species from congeners see the Discussion of *C. abulbus* sp. nov.

#### Cryptopygus abulbus sp. nov.

Taxon classificationAnimaliaEntomobryomorphaIsotomidae

B4A01B60-087A-5DF4-8D79-FBA0DBC37007

http://zoobank.org/EE9161B6-9521-407C-ADA5-1742B864573E

Figures 3 , 5 , 15–22

##### Type material.

Holotype and eighteen paratypes: South Africa • Western Cape, Stellenbosch, Jonkershoek Nature Reserve; 33.986883S, 18.955350E; 30 July 2009; C. Janion-Scheepers leg.; litter trap (J2_32), Holotype and eight paratypes deposited on four slides at SAMC, four paratypes on two slides deposited at SMNG, four paratypes on two slides deposited at MSPU, two paratypes on one slide at MNHN.

##### Diagnosis.

Without globular retractile bulb on Ant. IV. Organite on Ant. IV chili-like. 6+6 ocelli. Maxillary palp simple. Two sublobal hairs. Anterior side of manubrium without chaetae. Tenaculum with two chaetae.

##### Description.

**Figures 15–22.**
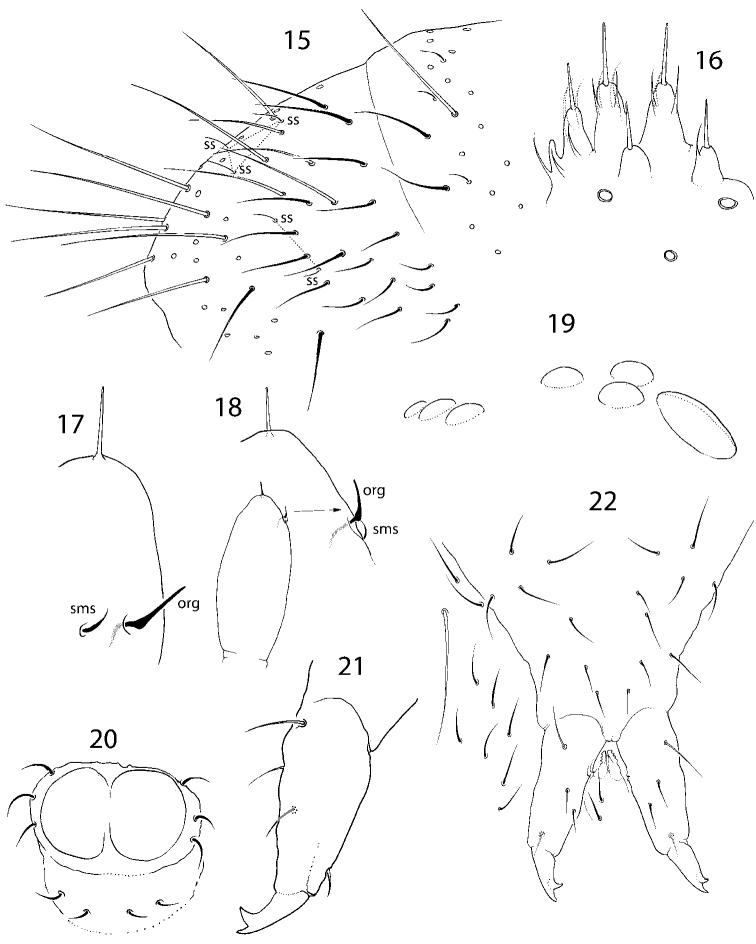
*C. abulbus* sp. nov. **15**Abd. IV and fused Abd. V and V **16** labial palp **17, 18** apical part of Ant. IV, different views **19** ocelli and PAO**20** ventral tube, ventral view **21** furca, lateral view **22** furcal area, ventral view. Abbreviations: org organit, sms subapical ms-chaeta, ss s-chaeta.

Body size 0.6–0.7 mm. Body with regular blue pigmentation, slender (Fig. 3). Abd. V well separated from Abd. IV and fused with Abd. VI (Fig. 15). Cuticle with orthogonal granulation. Ocelli 6+6 arranged as three in anterior and three in posterior group (Fig. 19). PAO more than twice as long as ocellus, 0.6–0.7 as long as width of Ant. I and 0.8–1.1 mm as long as inner unguis length. Maxillary outer lobe with two sublobal hairs, one individual with one sublobal hair on one side was found. Maxillary palp simple. Labral formula as 2/5,5,4. Labium with five usual papillae (*A–E*, Fig. 16) and labial formula as in *C. bulbus* sp. nov. Ventral side of head with 4+4 postlabial chaetae. Ant. I with eleven common chaetae, two ventral s-chaetae (s) and three small basal micro s-chaetae (bms), two dorsal and one ventral, Ant. II with three bms and one latero-distal s, Ant. III with one bms and five distal s (including one lateral). S-chaetae on Ant. IV weakly differentiated. Organite long, of chili-like shape, set apart from subapical micro s-chaeta (Figs 17, 18). Tip of antennae without retractile bulb.

Common chaetae slightly (under very high magnification) serrated at the posterior part of abdomen. S-formula as 4,3/2,2,2,3,5 (s), 1,0/1,0,0 (ms) (Figs 5, 15). Tergal s-chaetae much shorter than common chaetae and well distinguishable. Medial s-chaetae on Th. II-Abd. III situated in mid-tergal position. Macrochaetae smooth and short, 1,1/3,3,3 in number, medial ones on Abd. VI 1.6–2.0 times longer than dens and 2.8–4.1 times longer than mucro. Foil chaetae at the tip of abdomen absent. Axial chaetotaxy as 6–8,5/3,3,3,5–6. Th. I and II without chaetae, Th. III with 2+2 ventral chaetae.

Unguis without teeth. Empodial appendage 0.5–0.7 as in *C. bulbus* sp. nov. Tibiotarsi without additional chaetae on Leg I and II (21 chaetae), and with a few additional ones on Leg III, about 26 chaetae. Tibiotarsal tenent hairs clavate, 1,2,2 on Tibiotarsi 1,2,3. Ventral tube with 3+3 laterodistal and four posterior chaetae, anteriorly without chaetae (Fig. 20). Tenaculum with 4+4 teeth and two chaetae. Anterior furcal subcoxa with 5–7, posterior one with three chaetae. Anterior side of manubrium without chaetae, posterior side with 4+4 laterobasal and 8–9 chaetae on main part, without lateral chaetae. Dens short, without crenulation, with one rigid and short anterior and three posterior chaetae (Figs 21, 22). Mucro bidentate. Ratio manubrium : dens : mucro = 3.3–3.8 : 1.7–2.3 : 1.

##### Etymology.

The name is derived from the absence of apical bulb on Ant. IV to stress the difference from *C. bulbus* sp. nov.

##### Distribution and ecology.

Currently known from indigenous vegetation in the Jonkershoek Nature Reserve, Stellenbosch.

##### Discussion.

Unlike *C. bulbus* sp. nov., the new species has no antennal bulb. Nevertheless, the two species form a rather well-defined group differing from almost all congeners by a simple maxillary palp, two sublobal hairs, chili-shaped organite on Ant. IV, and the absence of chaetae on the anterior side of the manubrium. Concerning the last character, only *C. nivicolus* (Salmon, 1965) and *C. sverdrupi* Lawrence, 1978 also lack this pair of chaetae, which is common to other species of the genus. Both mentioned species are inhabitants of Antarctic polar deserts and can hardly be conspecific to *C. abulbus* sp. nov. found in dry sites in a subtropical climate. These two Antarctic species are very dark and have two clavate tenent hairs (vs. one in *C. abulbus* sp. nov.) on tibiotarsi I. In addition, *C. nivicolus* has no mucro (vs. present in the new species) while *C. sverdrupi* has very small PAO (more than twice longer than ocellus in *C. abulbus* sp. nov.). Recently, *Gressitacantha terranova* Wise, 1967 was moved to *Cryptopygus* (Greenslade, 2015) adding another *Cryptopygus* species without anterior chaetae on the manubrium. The differences between *C. abulbus* sp. nov. and *C. terranovus* are more numerous than those from *C. nivicolus* and *C. sverdrupi* (in furca, arms of abdomen, length of macrochaetae, and others).

#### Cryptopygus postantennalis sp. nov.

Taxon classificationAnimaliaEntomobryomorphaIsotomidae

313FA9A9-4DBF-54E9-AEAE-9133ED788E1B

http://zoobank.org/B6CDC3CA-C778-4B6B-A5EF-BE57FE61F6D6

Figures 23 , 26–31

##### Type material.

Holotype and three paratype: South Africa • Western Cape, Kogelberg Biosphere Reserve; 34.332650S, 18.950900E; 04 Oct. 2011; C. Janion-Scheepers leg.; Afromontane forest, litter/wood, Tullgren-Berlese extraction; RSA11_KOG007. Deposited on two slides at SAMC.

##### Other material.

South Africa • Western Cape, Haarwegskloof, Swellendam; 34.335968S, 20.325094E; 18 July 2017; O. Cowan leg. Deposited at MSPU.

##### Diagnosis.

1+1 ocelli. PAO very long and slender, with large inner denticles. MS-formula 1,0/0,0,1 (ms). All s-chaetae of Abd. V in one dorsal group. Anterior side of manubrium with 1+1 chaetae. Dens of medium length. Mucro tridentate.

##### Description.

**Figures 23–25.**
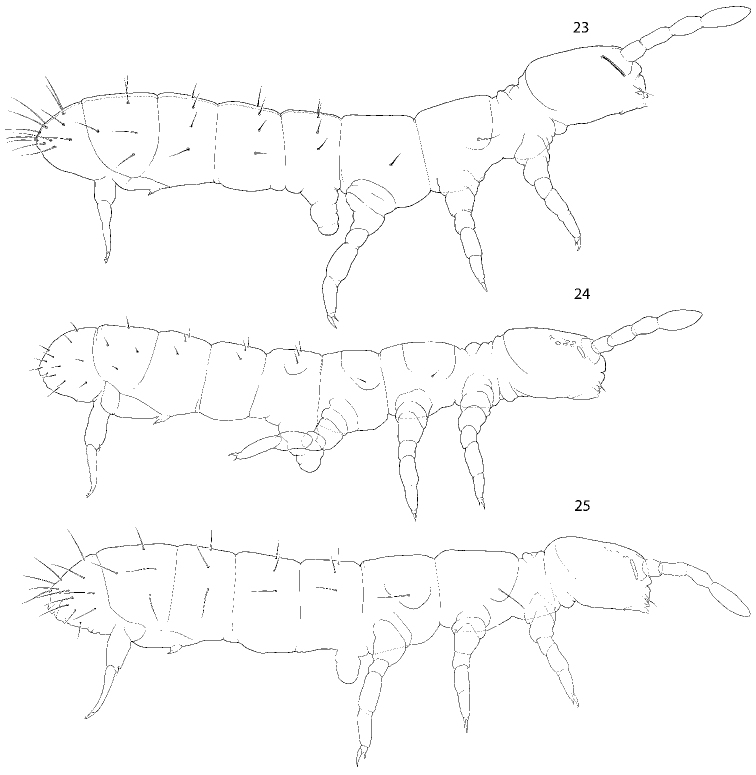
Appearance and macrochaetotaxy of *C. postantennalis* sp. nov. (**23**), *C. inflatus* sp. nov. (**24**), and *C. longisensillus* sp. nov. (**25**).

Body size 0.8 mm (only one adult female could be measured). Body mostly white, slender, with rare, scattered pigmentation and a distinct black eyespot (Fig. 23). Abd. V well separated from Abd. IV and fused with Abd. VI (Fig. 31). Cuticle with orthogonal and hexagonal granulation. One rudimentary ocellus, with a concentration of pigmentation. PAO very long and slender (Fig. 27), at least five times as long as ocellus, twice as long as width of Ant. I and 2.8 as long as inner unguis length. PAO constricted, with large “inner denticles”. Maxillary head with unmodified lamellae. Maxillary outer lobe with four sublobal hairs, maxillary palp bifurcate. Labral formula as 4/5,5,4, edge of labrum not reduced. Labium with five usual papillae (*A–E*), guard chaetae e7 present, three proximal and four basomedian chaetae. Ventral side of head with 4+4 chaetae. Ant. I with two ventral s-chaetae (s), distal one much shorter than proximal one, and three basal micro s-chaetae (bms), of which a long dorsal one (Fig. 28), Ant. II with three bms and one latero-distal s, Ant. III with one bms and five distal s (including one lateral), without additional s-chaetae. S-chaetae on Ant. IV weakly differentiated. Organite hook-like, close to subapical micro s-chaeta, which is long and bent (Fig. 29).

**Figures 26–31.**
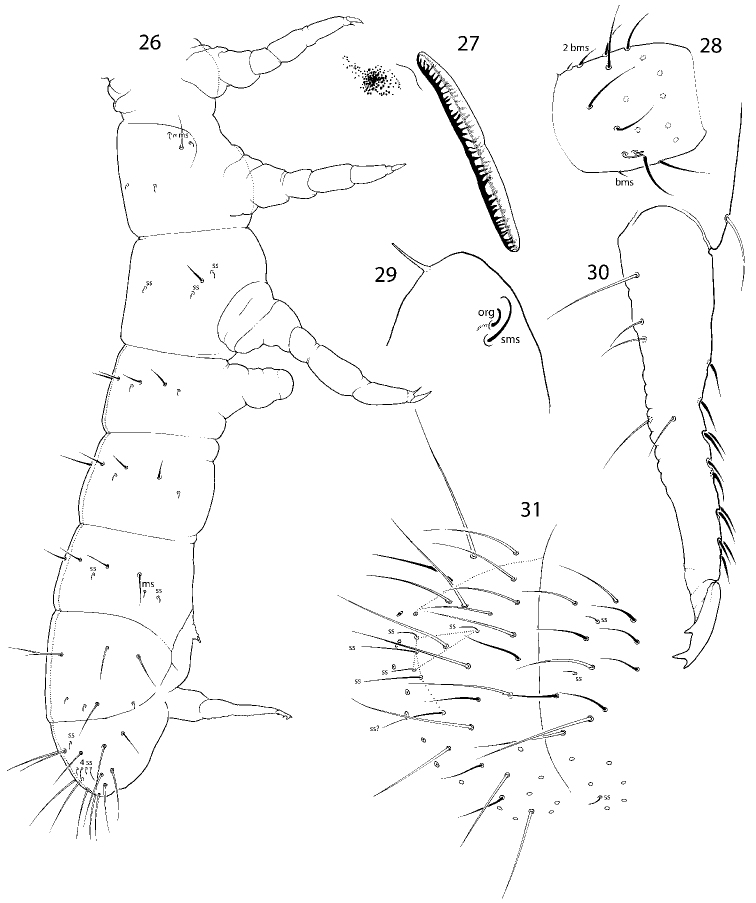
*C. postantennalis* sp. nov. **26** macrochaetotaxy and s-ms-pattern **27**PAO and eye area **28**Ant. I **29** apical part of Ant. IV **30** dens, lateral view **31**Abd. IV and V. Abbreviations: org organit, sms subapical ms-chaeta, ss s-chaeta, bms basal ms-chaeta, msms-chaeta.

Common chaetae often slightly serrated at the posterior part of Abd. V. S-formula as 4,3/2,2,2,3,5(6?) (s), 1,0/0,0,1 (ms) (Fig. 26). Ms on Abd. III as large as s-chaetae. Tergal s-chaetae much shorter than common chaetae and well distinguishable. Medial s-chaetae on Th. II-Abd. III situated in mid-tergal position. On Abd. V, three dorsal s-chaetae (triplet: al, accp1, accp2) short. Two lateral s-chaetae long, migrated to dorsal side and integrated to dorsal triplet (Fig. 31). One additional thin chaeta of unclear nature present on lateral side of Abd. V (notated as “ss?” in Fig. 31 and not shown in Fig. 26). Macrochaetae smooth and long, 1,1/3,3,3 in number, medial ones on Abd. VI more or less the same length as the dens and 3.8 times longer than mucro. Foil chaetae at the tip of abdomen absent. Axial chaetotaxy for abdomen 4–5,4–5,4–5, ca. 8 (difficult to observe in most individuals). All thoracic segments without ventral chaetae.

Unguis of normal shape, without teeth. Empodial appendage 0.5–0.6 as long as unguis. All tibiotarsi with additional chaetae: 2–3 chaetae on Legs I and II, and about five on Leg III. Tibiotarsal tenent hairs not clavate. Ventral tube with 3+3 laterodistal and 5–6 posterior chaetae, anteriorly without chaetae. Tenaculum with 4+4 teeth and two chaetae. Anterior furcal subcoxa with ten, posterior one with five chaetae (from one individual we could observe). Anterior side of manubrium with 1+1 chaetae. Dens of medium length, with crenulation, with 12 anterior and five posterior chaetae (Fig. 30). Mucro tridentate. Ratio manubrium : dens : mucro = 2.7–3.0 : 3.6–3.9 : 1.

##### Etymology.

The species is named after its remarkable PAO.

##### Distribution and ecology.

Known only from type locality and Swellendam, from indigenous vegetation (fynbos and Afromontane forest).

##### Discussion.

The new species has a unique PAO with large inner denticles visible at any magnification. All s-chaetae of Abd. V (al, accp1, accp2, accp3, accp4) are in one dorsal group, while in other species of *Cryptopygus* the triplet ‘al+accp1+accp2’ and duplet ‘accp3+accp4’ are placed in dorsal and lateral positions, respectively. The ms-formula 1,0/0,0,1 (vs. 1,1/1,1,1 or 1,0,1,0,0) is also unique.

#### Cryptopygus inflatus sp. nov.

Taxon classificationAnimaliaEntomobryomorphaIsotomidae

B5FF68BB-E4A9-5A6F-9054-336BC9BB7E53

http://zoobank.org/AE4D008B-A24F-446B-9C24-F90F1B3FA613

Figures 24 , 32–36

##### Type material.

Holotype and 21 paratypes: South Africa • Western Cape, Cederberg Wilderness Area, Wolfberg Cracks; 32.471507S, 19.278397E; 19 Feb. 2011; C. Janion-Scheepers leg.; litter, Tullgren extraction; RSA11_CED002. Holotype and three paratypes deposited on two slides at SAMC, six paratypes on three slides deposited at SMNG, four paratypes on two slides deposited at MSPU, and eight paratypes on four slides at NMHN. Ten paratypes in ethanol deposited at SAMC.

##### Other material.

South Africa • Western Cape, Cederberg Wilderness Area; 32.310167S, 19.175183E; Oct. 2008, C. Janion-Scheepers leg.; pitfall trap, RSA08_CED001.

Form with two chaetae on the basal part of the posterior side of the dens (see text below) from: South Africa, Northern Cape, Ezeljacht farm, 20 km from Sutherland, 32.4105S, 20.57747E, 1550m asl, 16 Jul. 2007, litter of shrub, C. Janion-Scheepers leg, RSA09_SUT001.

Form with three chaetae on the basal part of the posterior side of the dens (see text below) from: South Africa, Western Cape, Mont Rochelle Nature Reserve, Franschoek, 33.902967S, 19.158950E, 06 Oct. 2008, Litter trap with *Galenia* litter (MR510), C. Janion-Scheepers leg.

##### Diagnosis.

8+8 ocelli. Macrochaetae short. Anterior side of manubrium with 1+1 chaetae. Dens of medium length. Mucro tridentate.

##### Description.

Body size 0.6–0.9 mm. Body grey, Abd. V well separated from Abd. IV and fused with Abd. VI, slightly swollen (Figs 24, 33). Cuticle unmodified. Ocelli 8+8 (Fig. 36). PAO more than three times as long as ocellus, about as long as width of Ant. I and 1.8–2 times as long as inner unguis length. Maxillary head with unmodified lamellae. Maxillary outer lobe with four sublobal hairs, maxillary palp bifurcate. Labral formula as 3/5,5,4, edge of labrum not reduced. Labium with five usual papillae (*A–E*), guard chaetae e7 present, three proximal and four basomedian chaetae. Ventral side of head with 4+4 chaetae. Ant. I with two ventral s-chaetae (s) and three small basal micro s-chaetae (bms), two dorsal and one ventral (one dorsal large) (Fig. 34), Ant. II with three bms and one latero-distal s, Ant. III with one bms and five distal s (including one lateral), without additional s-chaetae. S-chaetae on Ant. IV weakly differentiated. Organite and subapical micro s-chaeta of normal shape, small and set together as normal.

**Figures 32–36.**
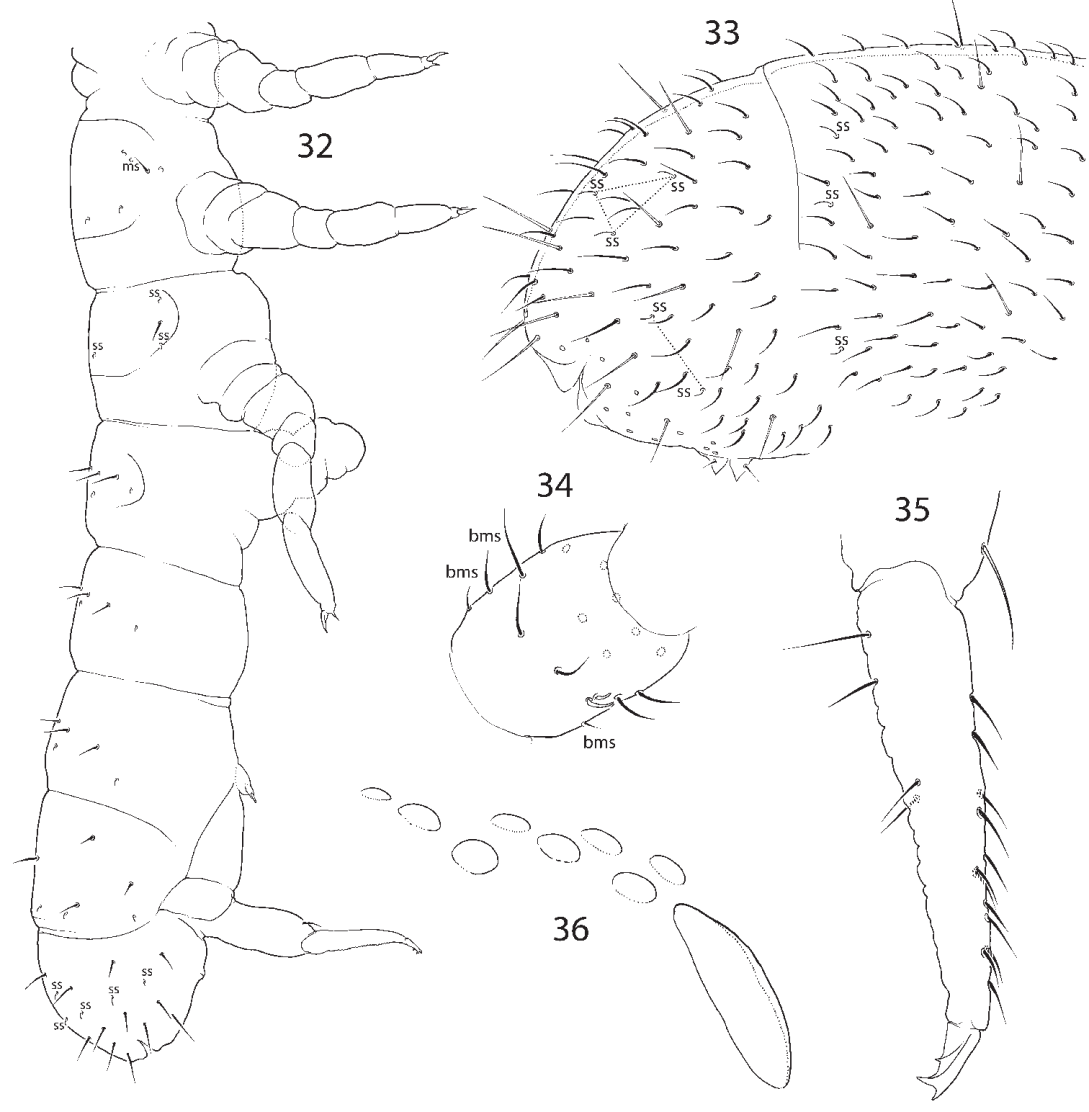
*C. inflatus* sp. nov. **32** macrochaetotaxy and s-ms-pattern **33**Abd. IV and fused Abd. V and VI **34**Ant. I **35** dens, lateral view **36** ocelli and PAO. Abbreviations: ss s-chaeta, bms basal ms-chaeta, msms-chaeta.

Common chaetae smooth. S-formula (Fig. 32) as 4,3/2,2,2,3,5 (s), 1,0/0,0,0 (ms). Tergal s-chaetae much shorter than common chaetae and well distinguishable. Medial s-chaetae on Th. II-Abd. III situated in mid-tergal position. On Abd. V all s-chaetae short subequal. Macrochaetae short, 1,1/3,3,3 in number, medial ones on Abd. VI 0.3–0.4 times longer than dens and 0.3–0.7 times longer than mucro. Foil chaetae at the tip of abdomen absent. Axial chaetotaxy as 8,7/4,4,4,7 (based on one individual). All thoracic segments without ventral chaetae.

Unguis of normal shape, without teeth. Empodial appendage about 0.6 as long as unguis. Tibiotarsi without additional chaetae on Leg I and II (21 chaetae), and with several chaetae on Leg III (>26). Tibiotarsal tenent hairs pointed. Ventral tube with 4+4 laterodistal and 5–6 posterior chaetae, anteriorly without chaetae. Tenaculum with 4+4 teeth and two chaetae. Anterior furcal subcoxa with 12–13, posterior one with 5–6 chaetae. Anterior side of manubrium with 1+1 chaeta, posterior side with 4+4 laterobasal and about 22 chaetae on main part, with a pair of lateral chaetae (Fig. 35). Dens normal, with crenulation, with 11–12 anterior and six posterior chaetae (three basal and two at middle and one subapical and very small). Mucro tridentate. Ratio manubrium : dens : mucro = 4.4–5.7 : 4.3–4.7 : 1.

##### Etymology.

The name is derived from the swollen posterior part of abdomen.

##### Distribution and ecology.

Known from mostly dry, mountainous areas, from the Northern Cape (Sutherland), the Cederberg Wilderness area, and Franschhoek (not as dry as previous two sites).

##### Discussion.

Considering all species in the *Cryptopygus* complex, the combination of 8+8 ocelli and tridentate mucro is unique. The only species resembling our species is Proisotoma (Isotomina) pseudominuta Schött, 1927, described from Cameroon. However, this species has clavate tenent hairs and a shorter dens, while *Cryptopygus inflatus* sp. nov. does not have clavate tenent hairs.

Some variation exists in the material examined and two forms of unclear status can be recognized; both have an ms on Abd. I (1,0/1,0,0) while *C. inflatus* sp. nov. does not. These two forms differ as follows:

a. Specimens from Sutherland (SUT002) have an ms and two chaetae on the basal part of the posterior side of the dens.

b. Specimens from Mont Rochelle (MR510) have an ms on Abd. I and three chaetae on the basal part of the posterior side of the dens like in *C. inflatus* sp. nov.

#### Cryptopygus longisensillus sp. nov.

Taxon classificationAnimaliaEntomobryomorphaIsotomidae

5E142D23-1B90-5C36-BEEA-E2404E628AAA

http://zoobank.org/EDBC3A2C-5B34-439A-BF2F-9C8868BF8991

Figures 25 , 37–41 , 42–46

##### Type material.

Holotype and six paratypes: South Africa • Northern Cape, Ezeljacht farm, 14 km from Sutherland; 32.4105S, 20.57747E; 1550 m asl; 16 July 2009; C. Janion-Scheepers leg.; shrub litter; RSA09_SUT002. Holotype and five paratypes on four slides deposited at SAMC, one paratype on one slide deposited at MSPU.

##### Other material.

Several specimens from South Africa • Western Cape, Jonkershoek Nature Reserve; 33.989350S, 18.957433E; 30 July 2009 and 12 Aug. 2010; C. Janion-Scheepers leg.; litter trap (32).

##### Diagnosis.

Anterior side of manubrium with 1+1 chaetae. Dens of medium length. Mucro tridentate. All s-chaetae of Abd. V elongated.

##### Description.

**Figures 37–41.**
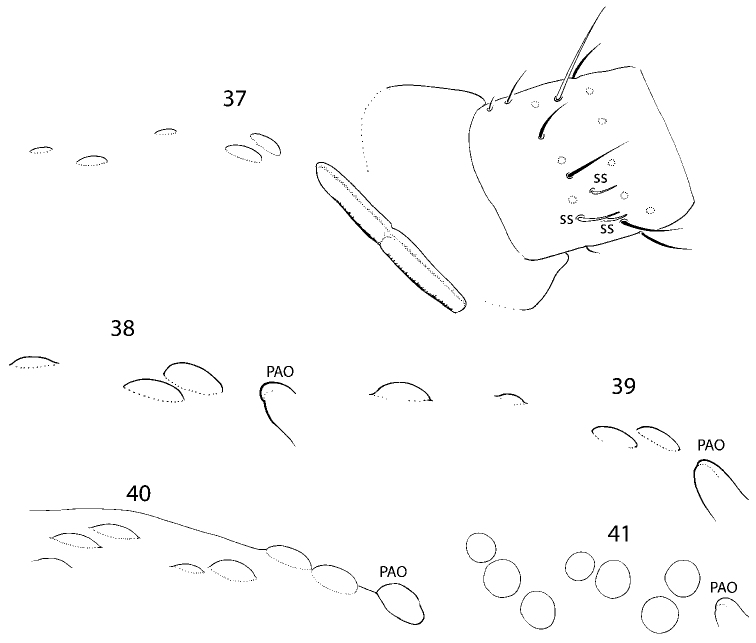
Variation of number of ocelli in *C. longisensillus* sp. nov. **37** 5+5 ocelli **38** 3+3 ocelli **39** 4+4 ocelli **40, 41** 7+7 ocelli.

Body size 0.9–1.3 mm (Fig. 25). Body pale, with scattered black granules of pigmentation, more concentrated on head, eye spots and posterior of trunk. Body tubular. Abd. V well separated from Abd. IV and fused with Abd. VI (Fig. 45). Cuticle with thin hexagonal primary granulation (“smooth”). Ocelli range from three to seven on each side (Figs 37–41) (see Discussion). PAO rather narrow, sharply constricted, with small inner denticles, longer than width of Ant. I (1.1–1.3) and inner unguis length (1.3–1.4). Maxillary head without modified lamellae. Maxillary outer lobe with four sublobal hairs, maxillary palp bifurcate. Labral formula as 4/5,5,4, edge of labrum not reduced. Labium with five usual papillae (*A–E*), guard chaetae e7 present, three proximal and four basomedian chaetae. Ventral side of head with 4–5+4–5 chaetae. Ant. I with three ventral s-chaetae (s) and two small basal micro s-chaetae (bms), dorsal and ventral, the former set together with long chaeta-like micro s-chaeta, Ant. II with three bms and one latero-distal s, Ant. III with one bms and six distal s (including two lateral), without additional s-chaetae. S-chaetae on Ant. IV weakly differentiated. Organite pin-like.

**Figures 42–46.**
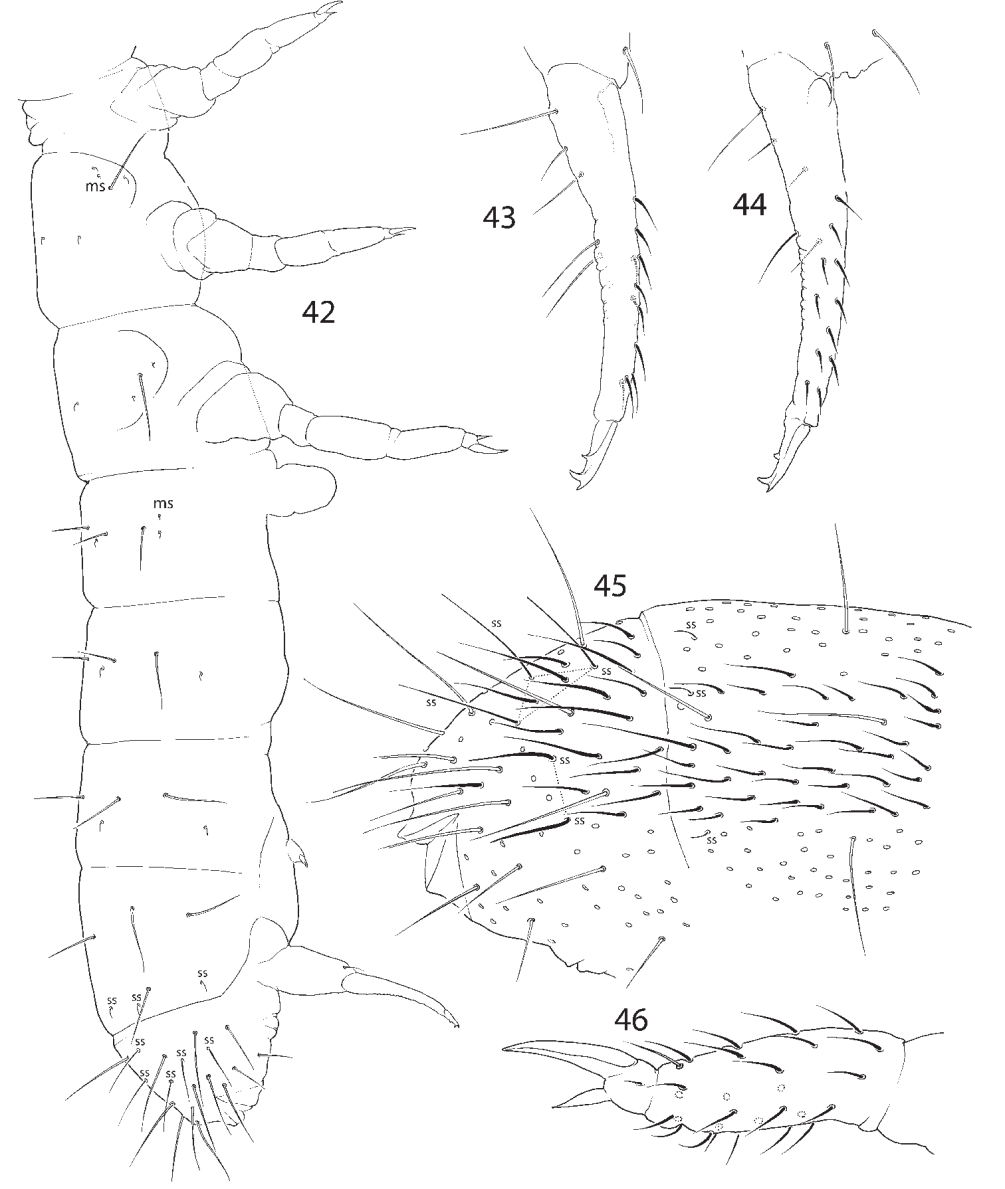
*C. longisensillus* sp. nov. **42** macrochaetotaxy and s-ms-pattern **43, 44** dens, lateral (**43**) and anterior (**44**) views **45**Abd. IV and fused Abd. V and VI **46** tibiotarsus and claw of Leg III. Abbreviations: ss s-chaeta, msms-chaeta.

Common chaetae rather long, smooth. S-formula as 4,3/2,2,2,3,5 (s), 1,0/1,0,0 (ms) (Fig. 42). Tergal s-chaetae short (apart from Abd. V) and well different from common chaetae. Medial s-chaetae on Th. II-Abd. III situated in mid-tergal position, on Abd. I–III between Mac1 and Mac2. Abd. V with five s-chaetae arranged with three dorsal ones (al, accp1, accp2), long and slender, and two lateral ones (accp3, accp4), long and slightly thickened so hardly distinguishable from common chaetae (Fig. 45). Macrochaetae smooth and long, 1,1/3,3,3 in number (Th. II-Abd. III), medial ones on Abd. VI 0.7–0.9 as long as dens and 2.8–3.7 times longer than mucro. Foil chaetae at the tip of abdomen absent. Axial chaetotaxy abundant 10–11,8–9/4–6,4–6,4–6. Thorax (incl. Th. III) without ventral chaetae.

Unguis of normal shape, without lateral and inner teeth. Empodial appendage about half as long as unguis. Tibiotarsi with additional chaetae, at whole with 23–24 ones on Leg I and II, and more than 25 on Leg III (Fig. 46). Tibiotarsal tenent hairs pointed. Adult males with stick-like thin spurs on Tibiotarsi III. Ventral tube with 3+3 laterodistal and six posterior chaetae, anteriorly without chaetae. Tenaculum with 4+4 teeth and a chaeta. Anterior furcal subcoxa with 13–15, posterior one with 4–6 chaetae. Anterior side of manubrium with 1+1 chaetae. Posterior side of manubrium with 12–14+12–14, including 5+5 on basolateral flaps. Dens with 10–12 anterior chaetae (Figs 43, 44). Posterior side of dens crenulated and with five chaetae (three basal and two at the middle). Mucro tridentate. Ratio manubrium : dens : mucro = 2.8–3.4 : 3.8–5.1 : 1.

##### Etymology.

The name is derived from the very long s-chaetae on Abd. V.

##### Distribution and ecology.

*Cryptopygus longisensillus* sp. nov. is currently known from Sutherland (Northern Cape) and Jonkershoek, Stellenbosch (Western Cape).

##### Discussion.

The species belongs to a group of forms having ocelli, tridentate mucro and rather long dens (*C. insignis* Massoud & Rapoport, 1968, *C. patagonicus* Izarra, 1972, *C. quadrioculatus* (Wise, 1970), *C. tricuspis* Enderlein, 1909) (Table 1). Two species, *C. insignis* and *C. tricuspis* have 1+1 chaetae (vs. 0+0 in *C. longisensillus* sp. nov.) on ventral side of Th. III and common s-chaetae (vs. elongated in new species) on Abd. V. Due to insufficient descriptions the morphology of *C. patagonicus* and *C. quadrioculatus* is less understood. Both species differ from *C. longisensillus* sp. nov. in fewer chaetae (eight and nine vs. 10–12) on anterior and more chaetae (six vs. five) on posterior sides of dens. *C. quadrioculatus* shows specific position of ocelli with one anterior and one posterior at a distance.

**Table 1.** Some key characteristics of *Cryptopygus* species having ocelli, tridentate mucro, and long dens.

Species	Ocelli	Chaetae on anterior side of dens	Chaetae on posterior side of dens	s-chaetae on Abd. V
*C. insignis*, Argentina	3+3	13	six	short
*C. patagonicus*, Argentina	5+5	eight	six	?
*C. quadrioculatus*, sub-Antarctic (South Georgia)	2+2	nine	six	?
*C. tricuspis*, sub-Antarctic (Kerguelen)	2+2	13	six	short
*C. longisensillus* sp. nov., South Africa	(3+3 to 7+7)	10–12	five	long

*Cryptopygus longisensillus* sp. nov. shows a unique pattern of s-chaetae on Abd. V not found before in the genus: all five s-chaetae elongated while s-chaetae of dorsal triplet thin, two lateral ones slightly thickened, representing a s-pattern of type “4” according to the classification of Potapov et al. (2017). The variability in the number of ocelli is unusual: among six individuals of one population we recorded variants with three, four, five, and seven ocelli. Two ocelli near the PAO are always well visible, while the other ones are only recognizable by weak swellings of the cuticle. The nature of this variability is not fully clear and calls for further study. The only similar case is known in *C. insignis* (fig. 4A in Massoud and Rapoport 1968). In the specimens from South Africa, Western Cape, Jonkershoek Nature Reserve, six posterior chaetae on dens were found. This variation also calls for further study.

### Morphological remarks on *C. insignis* Massoud & Rapoport, 1968

We had an opportunity to study two type specimens of this species kept in MNHN (Paris) labelled as “Lago Menendez 16.III.1959”. It was possible to observe the following characters: s-formula as 4,3/2,2,2,3,5 (s), 1,0/0,0,0 (ms), five s-chaetae on abd.V short, as common for the genus (unlike in *Cryptopygus longisensillus* sp. nov.); four prelabral chaetae, maxillary palp bifurcate; e7 present on labial palp; ventral tube with 4+4 laterodistal chaetae; Ant. I with three s-chaetae (s) and three basal micro s-chaetae, with long and short in dorsal group (like in *Cryptopygus longisensillus* sp. nov.).

#### Cryptopygus antarcticus

Taxon classificationAnimaliaEntomobryomorphaIsotomidae

complex

754E98EC-F524-565F-B8D4-EFF21DBE7EBF

##### Material.

South Africa • Western Cape, Kogelberg Nature Reserve; 34.331833S, 18.952467E; 04 Oct. 2011, C. Janion-Scheepers leg.; litter (*Mimetes* sp.), Tullgren extraction; RSAII_KOG010 • Western Cape, Orangekloof, Table Mountain National Park; 33.983883S, 18.403050E; 24 Oct. 2011, C. Janion-Scheepers leg.; indigenous vegetation litter, Tullgren extraction; RSAII_OK026 • Western Cape, Orangekloof, Table Mountain National Park; 33.975917S, 18.407667E; 24 Oct.2011; C. Janion-Scheepers leg.; indigenous vegetation litter, Tullgren extraction; RSAII_OK029 • South Africa, Western Cape, Table Mountain National Park, Cape Point, Platboom; 34.336017S, 18.447633E; 06 Aug. 2009; Tullgren extraction; RSA09_PEN008) • South Africa, Western Cape, Outeniqua, 33.887583S, 22.424067E, Afromontane Forest leaf litter Tullgren extraction (OUTF38), 14.ii.2013, A. Liu leg.; SAF-627 • Prince Albert: Swartberg South: Swartberg south slope; 12 March 2019; L. Deharveng & A. Bedos leg.; moss, moss on rock, Berlese; SAF-614, • Prince Albert, Swartberg North: Swartberg crest, 12 March 2019; L. Deharveng, C. Janion-Scheepers & A. Bedos leg.; moss, moss on rock, Berlese; SAF-401 • Constantia: Orange Kloof; 09 Jan. 2012; L. Deharveng & A. Bedos leg.; restio, litter, litter and humus, Berlese.

##### Diagnosis of the “antarcticus complex”.

4+4 to 6+6 ocelli. Manubrium with 1+1 chaetae on anterior side. Dens stout, not crenulated, with 4–6 anterior and 3–4 posterior chaetae. Mucro bidentate. Clavate tibiotarsal hairs present.

##### Distribution and ecology.

Currently known from the indigenous vegetation from the larger Table Mountain National park area, and the Outeniqua (George) area.

##### Discussion.

So far several subspecies and species resemble typical antarctic species *C. antarcticus* Willem, 1902 (*C. antarcticus maximus* Deharveng, 1981, *C. antarcticus travei* Deharveng, 1981, *C. antarcticus reagens* (Enderlein, 1909), *C. quinqueoculatus* Izarra, 1970, *C. hirsutus* Denis, 1931, *C. badasa* Greenslade, 1995, *C. araucanus* Massoud & Rapoport, 1968) and combine a group which is named “*antarcticus* complex” by us. In fact, they all almost fit to the strict diagnosis of the genus *Cryptopygus* proposed by Rusek (2002). This complex, often recorded just under the name ‘*C. antarcticus*’, is widely distributed in the sub-Antarctic (Deharveng 1981, Stevens et al. 2006) and less in temperate zones of the Southern Hemisphere (South America: Mari Mutt and Bellinger 1990; Australia: Greenslade, 1994; New Zealand: Babenko and Minor 2015), with *C. hirsutus* extending into Costa Rica. In South Africa, we have also found the forms with clavate tenent hairs on the tibiotarsi and the characteristic ‘*antarcticus*-like’ furca and so then belonging to ‘*C. antarcticus*’ complex. They differ from typical *C. antarcticus* at least by having fewer ocelli (three anterior and one or two posterior) and lighter pigmentation. So far, the following forms were found:

- micro s-chaetae 10\000, 4+4 ocelli Kogelberg (RSAII_KOG010), Table Mountain (RSAII_OKO26), and Cape Point (RSA09_PEN008).
- micro s-chaetae 10\100, 4+4 ocelli RSAII_OKO29 (Table Mountain)
- micro s-chaetae 10\100, 5+5 ocelli. This form fits descriptions of C. quinqueoculatus (Patagonia) well. OUT_F_38 (Outeniqua)
- micro s-chaetae 11\111, 4+4 ocelli SAF-401 (Table Mountain), SAF-614; SAF-627.

Reliable taxonomical decision on their status cannot be made at present and preliminary study of all known and unknown forms is underway.

#### Isotominella

Taxon classificationAnimaliaEntomobryomorphaIsotomidae

Delamare Deboutteville, 1948

CEC58D30-A28F-51F9-A820-6168EE26C3C2

##### Type species.

*Isotominella geophila* Delamare Deboutteville, 1948

##### Diagnosis.

The genus belongs to the *Cryptopygus* complex. Ocelli absent. Medial s-chaetae in posterior position from Th. II to Abd. IV. Number of s-chaetae 3,3/2,2,2,2,3. Foil chaetae at the end of abdomen absent.

##### Discussion.

The genus *Isotominella* was described from Ivory Coast and was subsequently given a detailed diagnosis by Jordana et al. (2009) based on material of *I. geophila* from Algeria. The taxon was considered a member of the *Cryptopygus* complex and was said to differ from related genera mainly by the crenulation, which is developed only in the proximal half of the dens. According to Jordana et al. (2009), in other genera of the complex this crenulation is either absent (i.e., dens is “smooth”), or extends further along the posterior side of dens, as in *Hemisotoma* Bagnall, 1949.

In our view, the crenulation is a flexible character within the genera of the *Cryptopygus* complex and depends on the length of furca, which can vary highly within a large genus, e.g., in *Cryptopygus* s. str., and particularly, among its representatives in South Africa (Figs 14, 30, 35, 43). Two species of the genus *Isotominella*, *I. geophila* and *I. laterochaeta* sp. nov. share a remarkable s-chaetotaxy, particularly, the posterior position of medial s-chaetae on body tergites, a reduced s-formula (3,3/2,2,2,2,3), and differentiation of s-chaetae on Abd. V with two long dorsal and one short lateral s-chaetae. The mouth parts of the two species are uncommon: the terminal ‘sensilla’ of papilla A and B are rod-like, the number of basolateral chaetae of labium are increased, the labrum has two prelabral chaetae, the number of sublobal hairs of maxillary outer lobe is reduced (three in the new species and two in *I. geophila*), and the maxillary head is modified. The presence of 7–9 basolateral chaetae on the labium (vs. commonly five as determined by Fjellberg 1999) in *I. laterochaeta* sp. nov. have not been recorded so far for Isotomidae. This remarkable feature is less pronounced in *I. geophila* in which this number is variable (five or six). Six basolateral chaetae on the labium were found in the genus *Pauropygus* (Potapov et al. 2013) in *P. projectus*Potapov et al., 2013, *P. caussaneli*, *P. pacificus*Potapov et al., 2013, which also shows the posterior position of the s-chaetae on tergites. The two genera share other important characters and are probably closely related (Potapov et al. 2013). Labral chaetae are normally pressed to the labrum in Isotomidae, while they are projected forward in the new species (the character is unclear for *I. geophila*). *Isotominella* also resembles the blind genera *Cylindropygus*Deharveng et al., 2005 (Europe) and *Dagamaea* Yosii, 1965 (East Asia and North America) but reliably differs by the posterior (vs. mid-tergal) position of the s-chaetae on the body tergites. A table to compare all the genera of the complex *Cryptopygus* is given by Jordana et al. (2009).

#### Isotominella laterochaeta sp. nov.

Taxon classificationAnimaliaEntomobryomorphaIsotomidae

55D5CB77-8C39-50DB-9A2A-997D716C58B0

http://zoobank.org/DD3D202C-F6D0-4912-A1BE-DFD11557771B

Figures 47–53 , 54–59

##### Type material.

Holotype and eight paratypes: South Africa • Western Cape, Platboom, Cape Point National Park; 34.336017S, 18.447633E; 14 Nov. 2010; L. Deharveng and A. Bedos leg.; soil; SAF 318. Holotype and two paratypes on three slides deposited at SAMC, three paratypes on three slides at NMHN, and three paratypes on three slides at MSPU.

##### Diagnosis.

Blind. Labium with 7–9 basolateral chaetae. Chaetae on ventral side of Th. III present. Labial palp with 16 guard chaetae. Three sublobal hairs on maxillary outer lobe. Anterior side of manubrium with 9–10+9–10 chaetae. Dens with 12–14 anterior and four posterior chaetae. Mucro bidentate.

##### Description.

**Figures 47–53.**
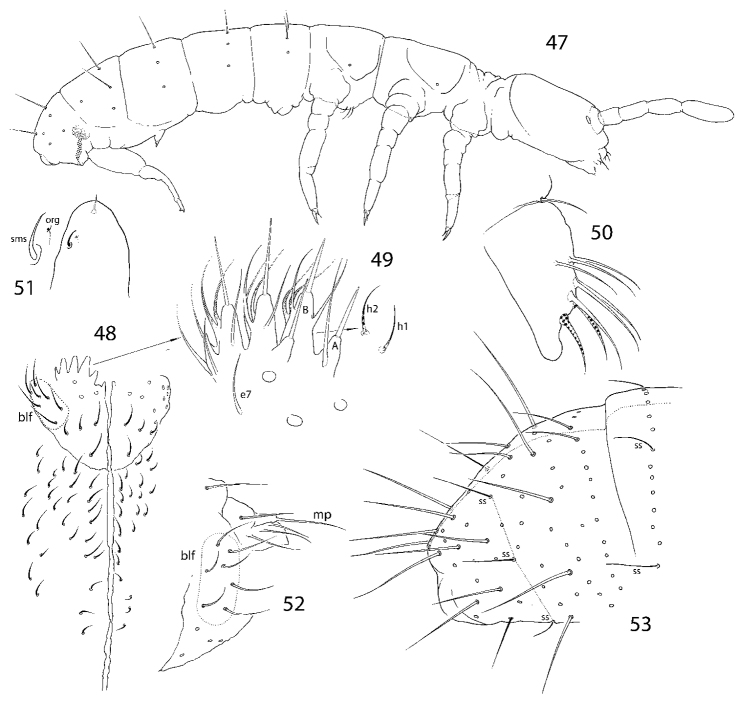
*Isotominella laterochaeta* sp. nov. **47** appearance and macrochaetotaxy (some macrochaetae lost) **48** ventral side of head **49** labial palp (hypostomal chaetae shown separately) **50** labrum, lateral view (chaetae of distal row marked, three, three, and two chaetae shown for proximal, middle, and distal rows, respectively) **51** apical part of Ant. IV **52** basal parts of labium and maxillary outer lobe, lateral view **53** posterior edge of Abd. IV and fused Abd. V and VI. Abbreviations: A and B papillae of labial palp, blf basolateral field of labium, e7 guard chaeta e7, mp maxillary palp, h1 and h2 hypostomal chaetae, org organit, sms subapical ms-chaeta, ss s-chaeta, msms-chaeta.

Body size 0.8–1.0 mm. White, without pigmentation, appearance as *Mucrosomia caeca* (Fig. 47). Mouth cone projected forward. Abd. V well separated from Abd. IV and fused with Abd. VI (Fig. 53). Cuticle “smooth”. Without ocelli. PAO small, not constricted, less than a half of width of Ant. I and 0.5–0.7 as long as inner unguis length. Maxillary head with slender lamellae and thin capitulum. Maxillary outer lobe with three sublobal hairs, maxillary palp simple (Fig. 52). Labral formula as 2/5,5,4, edge of labrum not reduced. Chaetae of labrum conspicuously projected forward (Fig. 50). Labral chaetae of middle and distal rows thicker than chaetae of proximal row. Inner chaetae of distal row shifted to more proximal position and integrated to middle row resulting the impression of 5,7,2 formula. Up to ten clypeal chaetae. Labium with five papillae (*A–E*), 16 guard chaetae (guard chaetae e7 present), and three proximal chaetae (Fig. 49). Terminal ‘sensilla’ of papillae *A* and *B* rod-like. All inner guard chaetae (b3, b4, d3, d4, e2, e3, e5, e6) strongly curved. With two curved and small accessorial hypostomal chaetae (h1, h2), main hypostomal chaeta (H) absent (Fig. 49). Basal part of labium with four basomedian and 7–9 basolateral chaetae (Figs 48, 52). Ventral side of head with numerous chaetae (up to 13 on one side along ventral line), with numerous chaetae at base of labium (Fig. 48). Normal s-chaetae and basal micro s-chaetae hardly differentiated on antennae, their number difficult to ascertain. Ant. I with many chaetae and at least two ventral s-chaetae (s) mixed with normal chaetae (or thinner s-chaetae). Ant. III with five distal s (including one lateral), without additional s-chaetae. S-chaetae on Ant. IV weakly differentiated. Organite small, rudimental, close to subapical micro s-chaeta, which is long and bent (Fig. 51).

**Figures 54–59.**
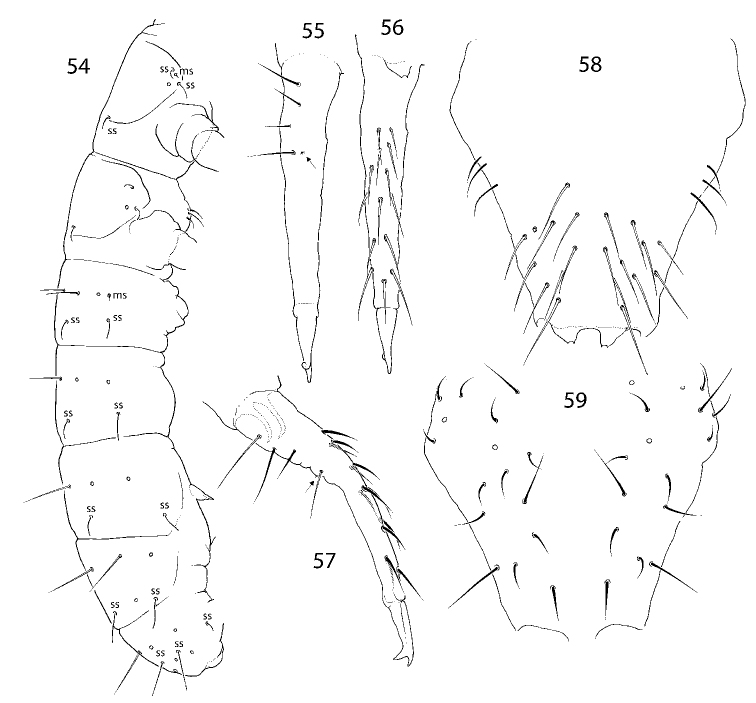
*I. laterochaeta* sp. nov. **54** macrochaetotaxy and s-ms-pattern **55–57** dens, posterior (**55**), anterior (**56**), and lateral (**57**) views **58, 59** manubrium, anterior (**58**) and posterior (**59**) views. Abbreviations: ss s-chaeta, msms-chaeta.

Common chaetae long. S-formula as 3,3/2,2,2,2,3 (s), 1,0/1,0,0 (ms) (Fig. 54). Tergal s-chaetae longer than common chaetae, less distinguishable on Th. II and III. Medial s-chaetae on Th. II-Abd. III situated in p-row of chaetae. On Abd. V, two dorsal s-chaetae (accp1, accp2) long, one lateral s-chaeta short (Fig. 53). Macrochaetae smooth and long, 1,1/3,3,3,4 in number. Macrochaetae on Abd. V longer than on Abd. VI: 0.8–0.9 and 0.6–0.7 as long as the dens and 3.3–3.5 and 2.3–2.8 times longer than mucro on Abd. V and VI, respectively. One thin macrochaetae (possibly additional s-chaeta) present in latero-ventral position on Abd. V. Foil chaetae at the tip of abdomen absent. Axial chaetotaxy for Th. II-Abd. IV 14–16,7–8/5–6, 5–6, 5–6, ca. 6. Th. III with 2–4+2–4 ventral chaetae.

Unguis of normal shape, without inner tooth and with two lateral teeth. Lateral teeth often asymmetrical in size and position. Empodial appendage thin, without lamellae, 0.6–0.7 as long as unguis. All tibiotarsi with many additional chaetae: Tibiotarsal tenent hairs not clavate. Ventral tube with 5–8+5–8 laterodistal and 6–9 posterior chaetae (with two larger in distal position), anteriorly without chaetae. Tenaculum with 4+4 teeth and two chaetae (rarely more). Anterior furcal subcoxa with 19–23, posterior one with 11–15 chaetae. Anterior side of manubrium with 9–10+9–10 chaetae arranged in two symmetrical group (Fig. 58), with distal pair thickened. Posterior side of manubrium with 11–14+11–14 chaetae on main part, 4+4 laterodistal and 2(3)+2(3) lateral chaetae (Fig. 59). Dens of medium length, with weak crenulation at the middle, with 12–14 anterior and four posterior chaetae (Figs 55–57). One minute chaeta-like process often seen close to distal chaeta. Mucro bidentate. Ratio manubrium : dens : mucro = 3.0–3.9 : 3.2–4.3 : 1.

##### Etymology.

The name is derived from its many lateral chaetae on the basal part of labium.

##### Distribution and ecology.

Known only from type locality (Cape Point National Park).

##### Discussion.

*Isotominella laterochaeta* sp. nov. and *I. geophila* (type species of the genus) differ by the number of anterior chaetae on the manubrium (9–10+9–10 vs. 1–4+1–4), chaetae on ventral side of Th. III (present vs. absent), number of guard chaetae on labial palp (16 vs. 13–14), and number of sublobal hairs (three vs. two) on maxillary outer lobe. Minute chaeta placed at the middle of posterior side of dens, numerous postlabial chaetae, and absence of hypostomal chaeta H are unique to *I. laterochaeta* sp. nov. The two last characters are unknown for *I. geophila*.

**Figure 60.**
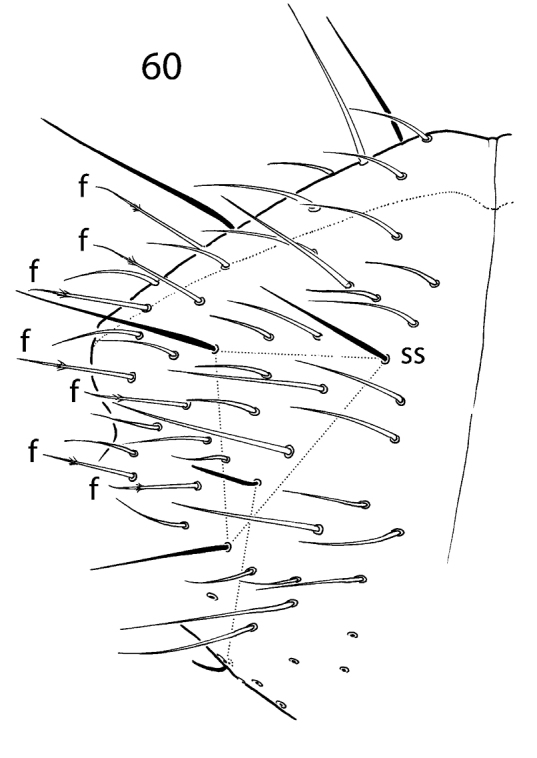
*Hemisotoma thermophila*, Abd. V–VI. Abbreviations: f foil chaeta, ss s-chaeta.

## General discussion and conclusions

The six new springtails from South Africa described here, five *Cryptopygus* and one *Isotominella*, bring the total number of Isotomidae to 25 species for the country. Our study is a first contribution to the knowledge of the rich fauna of *Cryptopygus* of this high endemism country. The five new species described are strongly dissimilar with each other and belong to different groups within the genus, suggesting different colonization events. In addition, local geographic speciation is suspected within several of these species, as reflected in the forms of unclear status presented in species discussions. They call for more detailed variability analyses on more extensive material in order to establish the taxonomical value of the morphological differences that were detected, to reconstruct the distribution patterns of the recognized forms, and to better understand the origin of this local geographical diversification.

## Supplementary Material

XML Treatment for Cryptopygus

XML Treatment for Cryptopygus bulbus

XML Treatment for Cryptopygus abulbus

XML Treatment for Cryptopygus postantennalis

XML Treatment for Cryptopygus inflatus

XML Treatment for Cryptopygus longisensillus

XML Treatment for Cryptopygus antarcticus

XML Treatment for Isotominella

XML Treatment for Isotominella laterochaeta

## References

[B1] Axelson WM (1900) Vorlaufige Mitteilung uber einige neue Collembolen-Formen aus Finnland. Meddelanden af Societas pro Fauna et Flora Fennica 26: 105–123. 10.5962/bhl.part.26726

[B2] Axelson WM (1903) Weitere Diagnosen uber neue Collembolen-Formen aus Finnland. Acta Societatis pro Fauna et Flora Fennica 25: 3–13.

[B3] Babenko A Minor M (2015) *Austrodontella monticola* sp. nov., a new species of Collembola from montane New Zealand. Zootaxa 3974: 122–128. 10.11646/zootaxa.3974.1.9 26249888

[B4] Bagnall RS (1949) Contribution towards a knowledge of the Isotomidae (Collembola) VII-XV. Annals and Magazine of Natural History 12: 81–96. 10.1080/00222934908653961

[B5] Barra JA (1997) Nouveaux Collemboles Entomobryomorphes des sables littoraux (partie terrestre) de la Province du Natal (Rép. Sud Africaine) (Insecta: Collembola). Journal of African Zoology 111: 465–480.

[B6] Börner C (1906) Das System der Collembolen nebst Beschreibung neuer Collembolen des Hamburger Naturhistorischen Museums. Mitteilungen aus den Naturhistorischen Museum in Hamburg 23: 147–188.

[B7] Deharveng L (1981) Collemboles des Iles Subantarctiques de l’Ocean Indien. Biologie des sols. Comité National Française des Recherches Antarctiques 48: 33–109.

[B8] Deharveng L Potapov M Bedos A (2005) *Cylindropygus ferox* gen. n., sp. n.: A new member of the *Cryptopygus* complex (Collembola, Isotomidae) from central France. Journal of Natural History 39: 2179–2185. 10.1080/00222930500061213

[B9] Delamare DC (1948) Recherches sur les Collemboles Termitophiles et Myrmécophiles (Écologie, Éthologie, Systématique). Archives de Zoologie Expérimentale et Générale 85: 261–425.

[B10] Denis JR (1931) Contributo alla conoscenza del Microgenton di Costa Rica II. Bollettino del Laboratorio di zoologia generale e agraria della R. Scuola superiore d’agricoltura in Portici 25: 69–170.

[B11] Enderlein G (1909) Die Insekten des antarctisches Gebietes. Deutsche Südpolar Expedition, 1901–1903 10: 361–458.

[B12] Fjellberg A (1999) The labial palp in Collembola. Zoologischer Anzeiger 237: 309–330.

[B13] Greenslade P (1994) Zoological Catalogue of Australia: Protura, Collembola, Diplura. (Ed. W.W.K. Houston). Melbourne, CSIRO Australia 22: 19–138.

[B14] Greenslade P (1995) Collembola from the Scotia Arc and Antarctic Peninsula including descriptions of two new species and notes on biogeography. Polskie Pismo Entomologiczne 64: 305–319.

[B15] Greenslade P (2015) Synonymy of two monobasic Anurophorinae genera (Collembola: Isotomidae) from the Antarctic Continent. New Zealand Journal of Zoology 38: 134–141. 10.1080/00779962.2015.1033810

[B16] Grinbergs A (1962) Uber die Collembolenfauna der Sowjetunion. II. Neue Collembolen aus der Tuvischen ASSR. Latvijas Entomologs 5: 59–67.

[B17] Izarra DC (1970) Tres nuevas especies de Colembolos de Siera de la Ventana (Provincia de Buenos Aires, Argentina). Physis 29: 393–397.

[B18] Izarra DC (1972) Fauna Collembologica de Isla Victoria (Provincia de Neuquen, Argentina). III. Familias Isotomidae y Entomobryidae Physis 31: 373–382. ca. South African Journal of Science 107: Art. #582. 10.4102/sajs.v107i11/12.582

[B19] Janion C Bedos A Deharveng L (2011b) The genus *Ectonura* Cassagnau, 1980 in South Africa (Collembola: Neanuridae: Neanurinae), with a key to South African Neanurinae. ZooKeys 136: 31–45. 10.3897/zookeys.136.1744 PMC3229287 22140347

[B20] Janion C Deharveng L Weiner WM (2013) Synonymy of *Spicatella* Thibaud, 2002 with *Delamarephorura* Weiner and Najt, 1999, and description of two new species (Collembola: Tullbergiidae). Raffles Bulletin of Zoology 61: 657–663. http://lkcnhm.nus.edu.sg/nus/pdf/PUBLICATION/Raffles%20Bulletin%20of%20Zoology/Past%20Volumes/RBZ%2061(2)/61rbz657-663.pdf.

[B21] Janion C D’Haese C Deharveng L (2012) A new species and first record of the genus *Triacanthella* Schaffer, 1897 (Collembola, Hypogastruridae) for Africa. ZooKeys 163: 57–68. 10.3897/zookeys.163.2298 PMC3253666 22303129

[B22] Janion C Bedos A Bengtsson J Deharveng L Jansen van Vuuren B Leinaas HP Liu A Malmström A Porco D Chown SL (2011a) Springtail diversity in South Afri Africa. South African Journal of Science 107: Art. #582. 10.4102/sajs.v107i11/12.582

[B23] Janion-Scheepers C Deharveng L Bedos A Chown SL (2015) Updated list of Collembola species currently recorded from South Africa. ZooKeys 503: 55–88. 10.3897/zookeys.503.8966 PMC4440272 26019671

[B24] Jordana R Hamra-Kroua S Baquero E (2009) Redescription of *Isotominella geophila* Delamare Deboutteville, 1948 from Algeria (Collembola, Entomobryomorpha, Isotomidae), A second world record for an Ivory Coast species. Zootaxa 2169: 63–68. 10.11646/zootaxa.2169.1.6

[B25] Lawrence PN (1978) *Cryptopygus sverdrupi* n. sp. A new species of Collembola (Isotomidae) from Sverdrupfjella, Antarctica, with notes on the related species in five genera. Norwegian Journal of Entomology 25: 51–55.

[B26] Mari Mutt JA Bellinger PF (1990) Flora & Fauna Handbook 5. A Catalog of the Neotropical Collembola, Including Neartic Areas of Mexico. Sandhill Crane Press, Gainesville, 237 pp.

[B27] Massoud Z Rapoport EH (1968) Collemboles Isotomides d’Amérique du Sud et de l’Antarctique. Biologie de l’Amérique Australe 4: 307–337.

[B28] McGaughran A Stevens MI Holland BR (2010) Biogeography of circum-Antarctic springtails. Molecular Phylogenetics and Evolution 57: 48–58. 10.1016/j.ympev.2010.06.003 20558307

[B29] Nicolet H (1842) Recherches pour servir a l’histoire des Podurelles. Neue Denkschriften der Allgemeinen Schweizerischen Gesellschaft für die gesammten Naturwissenschaften 6: 1–88.

[B30] Paclt J (1959) Collembola. South African Animal Life. Results of the Lund University Expedition in 1950–1951 6: 24–78.

[B31] Potapov M (2001) Synopses on Palaearctic Collembola (Vol. 3). Isotomidae. Abhandlungen und Berichte für Naturkunde Görlitz 73: 1–603.

[B32] Potapov MB Stebaeva SK (1994) *Sibiracanthella* and *Sahacanthella* new genera of Anurophorinae (Collembola, Isotomidae) with anal spines from continental Asia. Miscellania Zoologica 17: 129–139.

[B33] Potapov M Gao Y Deharveng L (2013) Taxonomy of the *Cryptopygus* complex. I. *Pauropygus*–a new worldwide littoral genus (Collembola, Isotomidae). ZooKeys 304: 1–16. 10.3897/zookeys.304.4083 PMC3689120 23794906

[B34] Potapov M Janion C Deharveng L (2011) Two new species of *Parisotoma* (Collembola: Isotomidae) from the Western Cape, South Africa. Zootaxa. 2771: 17–24. 10.11646/zootaxa.2771.1.2

[B35] Potapov M Janion-Scheepers C Deharveng L (2017) Taxonomy of the *Cryptopygus* complex. II. Affinity of austral *Cryptopygus* s.s. and *Folsomia*, with the description of two new *Folsomia* species (Collembola, Isotomidae) ZooKeys 658: 131–146. 10.3897/zookeys.658.11227 PMC5396566 28435389

[B36] Rusek J (2002) Do we have *Cryptopygus*-representatives in Europe? Pedobiologia 46: 302–310. 10.1078/0031-4056-00137

[B37] Salmon JT (1965) Two new genera of Antarctic Collembola. Pacific Insects 7: 468–472.

[B38] Schött H (1927) Kamerunischen Collembolen. Linköping 5: 1–40.

[B39] Stevens MI Greenslade P Hogg ID Sunnucks P (2006) Examining southern hemisphere springtails: could any have survived glaciation of Antarctica? Molecular Biology and Evolution 23: 874–882. 10.1093/molbev/msj073 16326749

[B40] Thibaud JM (1996) Étude des Collemboles (Hexapoda) interstitiels des sables littoraux de Mauritanie. Annales de la Société entomologique de France (N.S.) 32: 475–479.

[B41] Wahlgren E (1906) Antarktische und Subantarktische Collembolen gesammelt von der Schwedischen Sudpolarexpedition. Wissenschaftliche ergebnisse der Schwedischen sudpolarexpedition 1901–1903 5: 1–22.

[B42] Willem V (1902) Les collemboles recueillis par l’expédition antarctique belge. Annals of the society for Entomology of Belgium 45: 260–262.

[B43] Wise KAJ (1967) Collembola (Springtails). Antarctic Research Series 10: 123–148. 10.1029/AR010p0123

[B44] Wise KAJ (1970) Collembola of South Georgia. Pacific Insects Monograph 23: 183–208.

[B45] Yosii R (1965) On some Collembola of Japan and adjacent countries. Contributions from the Biological Laboratory Kyoto University 19: 1–71.

